# Editorial: Spotlight on the role of antibodies in viral clearance and tumour suppression

**DOI:** 10.3389/fmed.2024.1472625

**Published:** 2024-08-16

**Authors:** Maozhou He, Pengwei Ren, Heyu Chen, Yuanzhi Chen

**Affiliations:** ^1^School of Pharmaceutical Science and Technology, Hangzhou Institute for Advanced Study, University of Chinese Academy of Sciences, Hangzhou, China; ^2^Department of Surgery, Yale University School of Medicine, New Haven, CT, United States; ^3^AstraZeneca, Gaithersburg, MD, United States; ^4^State Key Laboratory of Vaccines for Infectious Diseases, Xiang An Biomedicine Laboratory, School of Life Sciences and School of Public Health, Xiamen University, Xiamen, China

**Keywords:** antibody therapy, antibody engineering, viral clearance, immunotherapy, tumor suppression

In an era where global health challenges such as viral infections and cancer continue to affect millions, understanding and innovating in the field of antibody therapeutics is more crucial than ever. As editors of this Research Topic, it's our pleasure to summarize the main findings and perspectives detailed within each of the accepted articles. In the dynamic landscape of medical research, the development and utilization of antibodies stand out as a cornerstone of therapeutic innovation, particularly in the fields of oncology. From a total of nine submissions received for this Research Topic, five exceptional articles were selected for publication. These articles delve into the latest advancements in antibody research, highlighting novel strategies that harness the precision of these biomolecules to combat complex diseases such as cancer and viral infections.

The article by Liu, Luan et al., presenting original research on a novel nanobody-based HER2-targeting antibody, introduces a groundbreaking approach to overcoming resistance in cancer therapy. Their findings not only showcase the potential of nanobodies in enhancing tumor targeting but also underscore the importance of innovative molecular engineering in developing more effective cancer treatments. This Research Topic sets the stage for future studies to explore how different therapeutic agents can work together and to understand the mechanisms behind resistance in targeted therapies.

Review articles in this Research Topic further enrich our understanding of antibody applications. Shao et al. explore the regulatory effects of growth differentiation factor 11 (GDF11), a protein with significant implications in tissue regeneration and disease therapy. Their comprehensive review elucidates the potential of GDF11 in clinical settings, providing a critical assessment of its therapeutic prospects and highlighting the challenges that lie ahead in its development as a drug.

In another review, the focus shifts to the Trop2 antigen, as discussed by Liu, Li et al.. Their work not only details the structural nuances of Trop2 but also evaluates the therapeutic efficacy of targeting this antigen in various cancers, emphasizing the need for safer and more effective antibody-drug conjugates (ADCs).

The clinical development of ADCs for non-small cell lung cancer therapy, as reviewed by Liu, Deng et al., marks a significant contribution to this Research Topic. The article reviews recent progress in ADCs, a class of biopharmaceuticals that has shown promising results in clinical trials, reflecting on the successes and limitations of these therapies in the context of resistance mechanisms.

Finally, the review by Dou et al. on the cellular composition of the tumor microenvironment in breast cancer provides insights into how cellular interactions within tumors can influence treatment outcomes. Their analysis underscores the importance of understanding the tumor microenvironment for predicting therapeutic efficacy, suggesting new avenues for research and antibody-based treatment optimization.

Each article in this Research Topic not only contributes to our knowledge base but also prompts further inquiry into the multifaceted roles of antibodies in disease management. By examining both the molecular intricacies and clinical applications of antibodies, this Research Topic of articles encourages a holistic view of the challenges and opportunities within antibody research. As we continue to explore the potential of these versatile molecules, it is clear that interdisciplinary efforts and innovative thinking will be crucial in harnessing their full therapeutic potential. The insights gained from this Research Topic underscore the pivotal role of antibodies in shaping the future of medical treatment, promising new strategies to combat some of the most challenging diseases of our time.

The editorial team is profoundly grateful to all the authors and reviewers for their substantial contributions to this Research Topic. We hope that the pioneering research presented here will serve as a valuable resource for furthering developments in the fields of viral and cancer therapeutics, inspiring ongoing innovation and collaboration in the scientific community.

